# Evaluation of the novel three lipid indices for predicting five- and ten-year incidence of cardiovascular disease: findings from Kerman coronary artery disease risk factors study (KERCADRS)

**DOI:** 10.1186/s12944-023-01932-x

**Published:** 2023-10-05

**Authors:** Alireza Jafari, Hamid Najafipour, Mitra Shadkam, Sina Aminizadeh

**Affiliations:** 1 Physiology Research Center, Institute of Neuropharmacology, Kerman University of Medical Sciences, Kerman, Iran; 2 Cardiovascular Research Center, Institute of Basic and Clinical Physiology Sciences, Kerman University of Medical Sciences, Kerman, Iran; 3 Endocrinology and Metabolism Research Center, Institute of Basic and Clinical Physiology Sciences, Kerman University of Medical Sciences, Kerman, Iran; 4 Faculty of Social Sciences, Shahid Bahonar University, Bulvd. 22 Bahman, Kerman, Iran; 5 Cardiovascular Research Center, Department of Physiology and Pharmacology, Afzalipour Medical Faculty, Kerman University of Medical Sciences, Blvd. 22 Bahman, Kerman, Iran

**Keywords:** Lipid accumulation product, Triglyceride-glucose index, Visceral adiposity index, Cardiovascular disease, Incidence

## Abstract

**Background:**

Data are limited on the relationship between cardiovascular disease (CVD) and the combinational indices of lipid accumulation product (LAP), triglyceride-glucose index (TyG), and visceral adiposity index (VAI). The association of these novel indices with the 5- and 10-year incidence of CVD was assessed.

**Method:**

A total of 1888 and 1450 healthy adults aged between 15 and 75 years (out of the 5895 participants of the KERCADR study, 2012) were followed for five and ten years, respectively. Baseline LAP, TyG, and VAI were calculated and logistic regression models were used to assess their relationship with the incidence of CVD in the two follow-up periods. Also, the predictive performance of these three indices was analyzed using the area under ROC curve (AUC) for the development of CVD compared with traditional single indices.

**Results:**

In the 5- and 10-year follow-ups, 399 and 476 CVD cases (21.1% and 32.8%) were documented, respectively. For the 5-year CVD risk, the adjusted odds ratio (AOR, 95% CI) was LAP (2.24 [1.44, 3.50]), VAI (1.58 [1.08, 2.33]), and TyG (1.57 [1.02, 2.42]). For the 10-year CVD risk, the AOR was LAP (1.61 [1.04, 2.49]), TyG (1.57 [1.02, 2.41]), and VAI (1.41 [0.96, 2.09]). In both periods and sexes, LAP had the best performance with the highest AUCs (0.644 and 0.651) compared to the other two indices and compared to the traditional single indices (e.g., BMI, LDL, etc.).

**Conclusion:**

Overall LAP, TyG, and VAI were better CVD risk predictors compared to the traditional single risk factors, with LAP showing the strongest predictive power for the incidence of CVD.

**Supplementary Information:**

The online version contains supplementary material available at 10.1186/s12944-023-01932-x.

## Introduction

Cardiovascular diseases (CVDs) are among the most important causes of death and disability worldwide, especially in developing countries [1]. Moreover, about half of the deaths in Iran are caused by CVDs, which continue to rise annually [2]. Evidence suggests that obesity, insulin resistance (IR), and dyslipidemia are known controllable risk factors in CVD development, which means effective screening strategies are required for them. In recent years, several novel anthropometric lipid markers, including lipid accumulation product (LAP) [3], triglyceride-glucose (TyG) index [4], and visceral adiposity index (VAI) [5–7], have been proposed as new CVD risk factors along with total cholesterol (TC), LDL-c, low HDL-c, and TG. However, the discriminatory accuracy and cutoff values of these novel risk factors are not well established.

LAP represents excess fat accumulation based on a combination of fasting triglyceride levels and waist circumference (WC) [8]. Studies have reported that LAP performs better than commonly used measures, such as BMI, WC, and waist-to-hip ratio (WHR), in predicting CVD.

It is believed that visceral fat plays a more important role in IR, impaired glucose tolerance, lipid metabolism, and blood pressure (BP) regulation compared to general and central obesity [10–12]. Amato et al. proposed VAI, a synthetic index that indirectly expresses visceral fat by combining anthropometry and laboratory lipid parameters, as a new marker [13].

TyG index has emerged as a promising surrogate marker for IR based on fasting blood glucose and triglyceride levels, offering a simple and cost-effective alternative to the more costly hyper-insulinemic-euglycemic clamp (HEC) technique [9].

A valid cut-off value is crucial for risk factor assessment in clinical use. However, there are limited data on the cut-off values of LAP, TyG, and VAI indices for identifying CVD. In Barzegar et al.‘s study, the cut-off value of TyG-index for CVD incident was determined to be 9.03 [4].

To date, few studies have investigated the relationship between LAP, TyG, and VAI, and CVD risk prediction in Iran. These studies were conducted in Tehran [3, 4, 6], Mashhad [10], and Ravansar [7]. However, no studies have been conducted in the Southern area of the country. Furthermore, no previous study has evaluated the effectiveness of LAP, TyG, and VAI comparatively in predicting CVD risk. Accordingly, due to shortage of sufficient information and cultural, nutritional, and lifestyle differences among various ethnic and socioeconomic groups, this study aimed to investigate the predictive power and cut-off value of these novel combinational lipid indices in the incidence of CVDs and compare their capability in long-term prediction of CVD incidence with traditional single indices (BMI, WC, TC, LDL-c, HDL-c, and TG) in the southeastern population of the country.

## Methods

### Study population

**Fig. 1:**
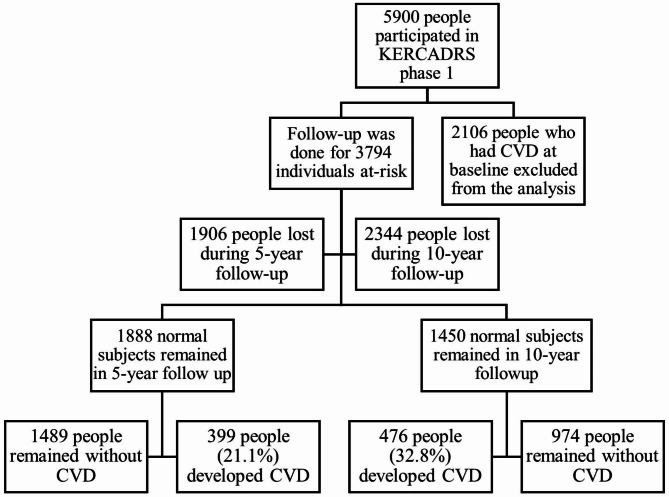
Flowchart outlining participants enrollment in the study

The Kerman coronary artery disease risk factors study (KERCADRS) is a prospective cohort conducted in Kerman, the largest city in southeastern Iran, with a population of 765,000. In 2012, 5895 participants (aged 15–75 years) were recruited in the first phase of KERCARDS. Of these, 1888 individuals participated again in the second (2017) and 1450 individuals participated in the third (2022) phases (Fig. 1). The follow up data of these individuals were analyzed in the present study. Written informed consent was obtained from participants in the KERCADR study. Approval for the study protocol was provided by the Ethics Committee of Kerman University of Medical Sciences (ethics code: IR.KMU.REC.1401.017) in accordance with the Declaration of Helsinki.

### Data collection and measurement

KERCADRS participants were selected using a one-stage cluster sampling method from 250 postal codes, which were randomly selected among the city postal codes. Participants’ demographic characteristics (age, sex, education, smoking, opium consumption), CVD risk behaviors, and medical history were assessed by physicians or trained personnel through physical examination or face-to-face interviews. Physical activity was classified as metabolic equivalents of task (METs) weekly values of < 1500 (low), 1500–3000 (moderate), or > 3000 (intense) units using the global physical activity questionnaire (GPAQ) [11]. Body weight, height, and WC were measured while the subject was standing without shoes. BP was measured after 10 min rest in sitting position, and medical history was taken by a physician. Following 10–12 h overnight fasting, a blood sample was taken to measure serum lipids and fasting blood glucose (FBG) using the spectrophotometric enzymatic methods (ELISA). LDL was calculated based on the Friedewald formula (LDL-C = TC − [HDL-C + TG / 5]). More details about the KERCADRS methodology can be found elsewhere [12].

### Definitions

The following formulas were used for calculating LAP, TyG, and VAI values in males and females:

$$LAP=\left[\left(WC-65\right)\times TG\right]\ for\, men$$

$$or\, \left[\left(WC-58\right)\times TG\right]\ for\, women$$ [8].

$$TyG=ln\left(\frac{TG \times FBG}{2}\right)$$ [9].

$$VAI=\left[\left(\frac{WC}{39.68+\left(1.88 \times BMI\right)}\right)\times\left(\frac{TG}{1.03}\right)\times\left(\frac{1.31}{HDL}\right)\right]for\, men$$

$$or\, \left[\left(\frac{WC}{36.58+(1.89\times BMI)}\right)\times\left(\frac{TG}{0.81}\right)\times\left(\frac{1.52}{HDL}\right)\right]for\, women$$ [13].

where TG and HDL levels are in mmol/l and WC is in cm.

Hypertension (HTN) was defined as an individual having a systolic BP (SBP) ≥ 140 mmHg and/or a diastolic BP (DBP) ≥ 90 mmHg and/or receiving antihypertensive medications. CVD was defined as being hypertensive, having medical history of ischemic heart disease (IHD), heart failure, angina pectoris, stroke, MI, and/or the current use of medication prescribed by a physician for CVDs.

### Statistical analysis

Data were statistically analyzed using Stata 17.0 software (College Station, TX: Stata Corp LLC, USA). The normality of distribution for continuous variables was tested using the Kolmogorov-Smirnov test. Descriptive statistics were presented for continuous variables as mean ± standard deviation (SD) or median and 25th–75th percentile (P). Categorical variables were presented as number (%). ANOVA or chi-square test was used to compare baseline characteristics across CVD and non-CVD groups. The continuous variables of the three new indices were transformed into categorical variables by using quartile methods. Correlation analysis was performed to assess the correlation between the three new indices and CVD risk. Multivariable binary logistic regressions were used to assess the association between the three indices and CVD risk. We selected these confounders based on their associations with the CVD incidence or a change of more than 10% in effect estimate. We also did subgroup analyses according to sex. To determine the cut-off points predicting CVD incidence, we utilized Youden’s index (sensitivity + specificity − 1) and calculated the area under the curve (AUC (95% CI)). All tests were two-sided, and *P*-values < 0.05 were considered significant.

## Results

Participant characteristics (demographic, laboratory, and anthropometric data) according to CVD events in the 5- and 10-year follow up groups are shown in Table 1. The analysis of the data of the 1888 subjects in the 5-year follow-up group showed that their average age was 54.06 years, and 43.2% of them were men. Of the subjects, 1489 had not experienced CVDs and 399 (21.1%) had experienced CVDs. The results also showed that, participants with CVD were older (Mean 61.5 vs. 52.1 years) and had a higher BMI (27 vs. 25.1 Kg/m^2^), WC (88.8 vs. 82.2 cm), SBP (115.7 vs. 107.8 mmHg), and DBP (76.1 vs. 73.1 mmHg), (all *P* < 0.01, except for VAI with *P* < 0.05). No significant difference in smoking, education, and physical activity levels was observed between non-CVD and CVD participants. Table 1 also shows that, among the 1450 participants in the 10-year follow up group, the average age was 53.5 years, 43.4% were male, and 476 (32.8%) experienced CVDs during the ten years. Age (Mean 59.3 vs. 50.7 years), BMI (26.9 vs. 24.9 Kg/m^2^), WC (87.7 vs. 81.7), FBS (101.5 vs. 94.2 mg/dl), TC (202 vs. 187.2 mg/dl), TG (163 vs. 131.8 mg/dl), LDL (132.4 vs. 122.4 mg/dl), SBP (115 vs. 107 mmHg), DBP (75.7 vs. 72.8 mmHg), LAP (52 vs. 34.4), TyG (8.84 vs. 8.57), and VAI (3.39 vs. 2.69) were significantly higher in the CVD group compared to the non-CVD group (all *P* < 0.01). Furthermore, the level of university education (16.8% vs. 22.2%) was lower in the CVD group. Considering gender differences, in comparison to non-CVD males, CVD males had lower HDL levels. On the other hand, FBS, TC, TG, and LDL levels were significantly higher in CVD females compared to non-CVD females in the 5-year follow-up group (Supplementary Table 1). The men in the CVD group had lower physical activity levels compared to the non-CVD males in the 10-year follow-up group (Supplementary Table 2).

**Table 1:** Comparison of baseline characteristics of participants

Subgroups	5-Year follow-up (*n* = 1888)		10-Year follow-up (*n* = 1450)	
	Non-CVD group (n = 1489)	CVD group (n = 399)	Non-CVD group (n = 974)	CVD group (n = 476)
Age, mean (SD)	52.08 (13.73)	61.50 (12.52) ^‡^	50.67 (12.73)	59.33 (11.78) ^‡^
Male, n (%)	632 (42.44)	183 (45.86)	420 (43.12)	209 (43.91)
BMI (kg/m^2^)	25.07 (4.76)	26.96 (4.64) ^‡^	24.95 (4.84)	26.89 (4.33) ^‡^
WC (cm) mean (SD)	82.2 (11.6)	88.8 (11.7) ^‡^	81.7 (11.9)	87.7 (10.9) ^‡^
Education (Univ.) n (%)	297 (19.97)	72 (17.80) ^‡^	216 (22.21)	80 (16.84) ^‡^
Cigarette smoking, n (%)	156 (10.48)	44 (11.03)	93 (9.55)	54 (11.34)
Low physical activity n (%)	627 (42.11)	174 (43.61)	396 (40.66)	213 (44.75)
FBS (mg/dl)	95.96 (27.81) 91 [83, 101]	104.55 (39.3) ^‡^ 95 [85, 108]	94.22 (25.50) 90 [82, 100]	101.49 (31.25) ^‡^ 94 [86, 106]
TC (mg/dl)	189.86 (41.81)	199.15 (41.7) ^‡^	187.26 (41.51)	201.96 (40.07) ^‡^
TG (mg/dl)	136.82 (89.04) 114 [81, 165]	156.4 (84.3) ^‡^ 135 [99, 186]	131.80 (82.31) 114 [81, 165]	163.05 (98.58) ^‡^ 137.5 [99,197.5]
HDL-C (mg/dl)	38.96 (9.58)	37.94 (9.50)	38.84 (9.45)	38.20 (9.66)
LDL-C (mg/dl)	124.00 (35.06)	130.8 (34.4) ^‡^	122.42 (35.10)	132.40 (33.77) ^‡^
SBP (mm Hg)	107.81 (12.34)	115.7 (11.37) ^‡^	107.13 (12.61)	115.22 (12.14) ^‡^
DBP (mm Hg)	73.14 (7.17)	76.15 (6.17) ^‡^	72.81 (7.43)	75.77 (6.79) ^‡^
LAP	36.48 (35.08) 27.6 [13.0, 48,4]	51.09 (38.10) ^‡^ 42.3 [24.2, 65.2]	34.39 (32.92) 26.2 [12.0, 46.4]	52.04 (41.41) ^‡^ 41.5 [23.99, 66.4]
TyG	8.61 (0.62)	8.84 (0.60) ^‡^	8.57 (0.59)	8.84 (0.64) ^‡^
VAI	2.76 (2.91) 2.02 [1.29, 3.21]	3.21 (2.38) ^†^ 2.48 [1.60, 3.97]	2.61 (2.49) 1.95[1.16, 2.91]	3.39 (3.16) ^‡^ 2.47 [1.55, 4.14]

CVD, cardiovascular disease; FBS, fasting blood sugar; TC, total cholesterol; TG, triglyceride; HDL, high-density lipoprotein; LDL, low-density lipoprotein; SBP, systolic blood pressure; DBP, diastolic blood pressure; WC, waist circumference; LAP, lipid accumulation product; TyG, triglyceride glucose index; VAI, visceral adiposity index

Data are presented as Mean (SD) or Median [P25-75] for continuous measures, and n (%) for categorical measures

Significant levels of ^†^*P* < 0.01 and ^‡^*P* < 0.001 using ANOVA test

Multiple logistic regression analysis was used to assess the association between LAP, TyG, VAI, and CVD incidence, and the results are shown in Tables 2 and 3. When compared to the 1st quartile, the 5-year adjusted OR for LAP, VAI, and TyG for the 4th quartile was (2.24; 95% CI:1.44, 3.50; *P* < 0.01), (1.58; 95% CI: 1.08, 2.33; *P* < 0.05), and (1.57; 95% CI: 1.02, 2.42; *P* < 0.05), respectively (Table 2). In comparison, the 10-year adjusted OR for LAP, TyG, and VAI was (1.61; 95% CI: 1.04, 2.49; *P* < 0.05), (1.57; 95% CI: 1.02, 2.41; *P* < 0.05), and (1.41; 95% CI: 0.96, 2.09; *P* < 0.05), respectively (Table 3). Furthermore, the 5-year AOR across quartiles indicated a significant trend for LAP and VAI with CVD incidence (*P*_trend_ < 0.05 for both) (Table 2). In the context of the 10-year follow-up, only LAP showed a significant increase with CVD incidence across the quartiles (*P*_trend_ = 0.023) (Table 3).

**Table 2:** Association between LAP, TyG, and VAI quartiles with 5-year incidence CVD odds

Variables	Quartile numbers	CVD cases	Crude OR (95% CI)	Adjusted OR (95% CI)
LAP				
Q1 < 14.77	471	45	1.00 (as reference)	1.00 (as reference)
Q2 14.77–31.45	472	80	1.93 (1.31, 2.85)	1.21 (0.80, 1.85)
Q3 31.46–53.89	470	126	3.46 (2.40, 5.01)	1.86 (1.23, 2.81) ^‡^
Q4 > 53.89	470	147	4.31 (2.99, 6.20)	2.24 (1.44, 3.50) ^‡^
*P* for trend			< 0.001	< 0.001
OR Per 1 increment	1883	398	1.01 (1.01, 1.01)	1.01 (1.00, 1.01)
TyG				
Q1 < 8.21	471	55	1.00 (as reference)	1.00 (as reference)
Q2 8.21–8.61	470	103	2.12 (1.49, 3.03)	1.47 (1.00, 2.16)
Q3 8.62–9.04	473	103	2.11 (1.47, 3.01)	1.25 (0.84, 1.86)
Q4 > 9.04	468	137	3.13 (2.22, 4.42)	1.57 (1.02, 2.42) ^†^
*P* for trend			< 0.001	< 0.126
OR Per 1 increment	1882	398	1.77 (1.49, 2.12)	1.30 (1.00, 1.70)
VAI				
Q1 < 1.35	470	64	1.00 (as reference)	1.00 (as reference)
Q2 1.35–2.08	470	95	1.61 (1.14, 2.27)	1.28 (0.88, 1.86)
Q3 2.09–3.39	470	105	1.82 (1.30, 2.57)	1.27 (0.88, 1.84)
Q4 > 3.39	470	133	2.50 (1.80, 3.49)	1.58 (1.08, 2.33) ^†^
*P* for trend			< 0.001	0.03
OR Per 1 increment	1880	397	1.05 (1.01, 1.09)	1.06 (0.99, 1.13)

LAP, lipid accumulation product; TyG, triglyceride glucose index; VAI, visceral adiposity index; OR, odds ratio; CI, confidence interval; Q, quartile

Crude: Not adjusted for any variables

Adjusted: for age, sex, job, education, physical activity, smoking, total cholesterol, blood pressure, and LDL

*P* values are calculated using multivariable logistic regression

Significant level of † *P* < 0.05 and ‡ *P* < 0.01

**Table 3:** Association between LAP, TyG, and VAI quartiles, with 10-year incidence CVD odds

Variables	Quartile numbers	CVD cases	Crude OR (95% CI)	Adjusted OR (95% CI)
LAP				
Q1 < 16.75	363	61	1.00 (as reference)	1.00 (as reference)
Q2 16.76–38.16	360	105	2.04 (1.43, 2.91)	1.25 (0.84, 1.85)
Q3 38.16-158.32	362	141	3.16 (2.23, 4.47)	1.52 (1.02, 2.27) ^†^
Q4 > 158.32	363	169	4.31 (3.06, 6.08)	1.61 (1.04, 2.49) ^†^
*P* for trend			< 0.001	0.023
OR Per 1 increment	1448	476	1.01 (1.01, 1.01)	1.01 (1.00, 1.01)
TyG				
Q1 < 8.47	360	78	1.00 (as reference)	1.00 (as reference)
Q2 8.47–8.84	362	107	1.48 (1.06, 2.07)	1.01 (0.70, 1.49)
Q3 8.84–9.10	364	112	1.59 (1.14, 2.23)	0.85 (0.57, 1.25)
Q4 > 9.10	361	179	3.51 (2.54, 4.85)	1.57 (1.02, 2.41) ^†^
*P* for trend			< 0.001	0.098
OR Per 1 increment	1447	476	2.06 (1.72, 2.49)	1.20 (0.91, 1.57)
VAI				
Q1 < 1.50	362	78	1.00 (as reference)	1.00 (as reference)
Q2 1.50–2.48	361	121	1.84 (1.32, 2.56)	1.54 (1.07, 2.22) ^†^
Q3 2.48–3.65	361	118	1.77 (1.27, 2.47)	1.09 (0.75, 1.58)
Q4 > 3.65	362	150	2.85 (2.06, 3.95)	1.41 (0.96, 2.09)
*P* for trend			< 0.001	0.319
OR Per 1 increment	1446	477	1.11 (1.06, 1.16)	1.01 (0.94, 1.08)

LAP, lipid accumulation product; TyG, triglyceride glucose index; VAI, visceral adiposity index; OR, odds ratio; CI, confidence interval; Q, quartile

Crude: Not adjusted for any variables

Adjusted for age, sex, job, education, physical activity, smoking, total cholesterol, blood pressure, and LDL

*P* values are calculated using multivariable logistic regression

Significant level of †*P* < 0.05 and ‡*P* < 0.01

Tables 4 and 5 demonstrate the Pearson correlation coefficient between participants’ characteristics with LAP, TyG, and VAI in the 5- and 10-year follow-ups. The results show that there is a significant correlation, in both time periods, between these three indices and all variables except in relation to VAI for DBP. LAP, TyG, and VAI were not significantly correlated with smoking, education, and physical activity in the 5- and 10-year follow-ups (Tables 4 and 5).

**Table 4:** Correlation between LAP, TyG, VAI, and anthropometric and biochemical variables with the 5-year incident of CVD.

Variable	LAP	TyG	VAI
Age (years)	0.299*	0.349*	0.159*
Cigarette smoking	−0.035	−0.075	−0.036
Education	−0.066	−0.097	0.068
Physical activity	−0.081	−0.064	−0.072
BMI (kg/m^2^)	0.553*	0.333*	0.229*
WC (cm)	0.685*	0.440*	0.288*
FBS (mg/dl)	0.229*	0.527*	0.165*
TC (mg/dl)	0.413*	0.488*	0.264*
TG (mg/dl)	0.855*	0.855*	0.905*
HDL-C (mg/dl)	−0.361*	−0.423*	−0.532*
LDL-C (mg/dl)	0.278*	0.344*	0.156*
SBP (mm Hg)	0.252*	0.245*	0.110*
DBP (mm Hg)	0.188*	0.175*	0.102

BMI, body mass index; DBP, diastolic blood pressure; FBS, fasting blood sugar; HDL, high-density lipoprotein; LAP, lipid accumulation product; LDL, low-density lipoprotein; SBP, systolic blood pressure; TC, total cholesterol; TG, triglyceride; TyG, triglyceride-glucose index; VAI, visceral adiposity index, WC, waist circumference

*P < 0.001

**Table 5:** Correlation between LAP, TyG, VAI, and anthropometric and biochemical variables with the 10-year incidence of CVD.

Variable	LAP	TyG	VAI
Age (years)	0.304*	0.336*	0.166*
Cigarette smoking	−0.054	−0.088	−0.054
Education	−0.045	−0.058	0.056
Physical activity	−0.067	−0.032	−0.050
BMI (kg/m^2^)	0.556*	0.347*	0.243*
WC (cm)	0.690*	0.450*	0.308*
FBS (mg/dl)	0.233*	0.509*	0.169*
TC (mg/dl)	0.403*	0.478*	0.269*
TG (mg/dl)	0.858*	0.864*	0.906*
HDL-C (mg/dl)	−0.373*	−0.443*	−0.544*
LDL-C (mg/dl)	0.250*	0.319*	0.133*
SBP (mm Hg)	0.275*	0.275*	0.132*
DBP (mm Hg)	0.194*	0.186*	0.108

BMI, body mass index; DBP, diastolic blood pressure; FBS, fasting blood sugar; HDL, high-density lipoprotein; LAP, lipid accumulation product; LDL, low-density lipoprotein; SBP, systolic blood pressure; TC, total cholesterol; TG, triglyceride; TyG, triglyceride-glucose index; VAI, visceral adiposity index, WC, waist circumference

*P < 0.001

**Fig. 2:**
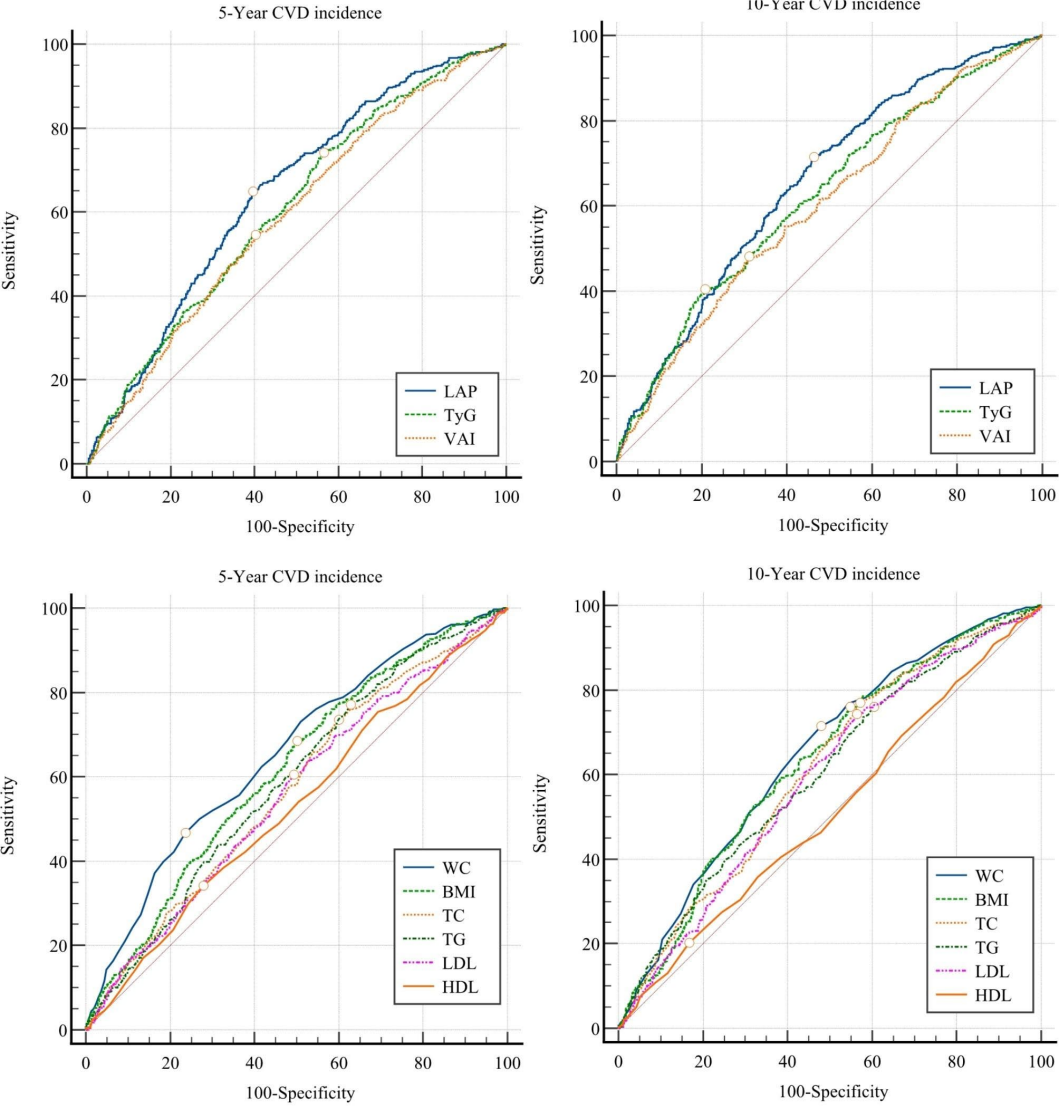
Comparisson of reciver operative charachteristic (ROC) curve of new (upper panels) and traditional (lower panels) CVD risk factors in 5-year (left) and 10-year (right) periods

The AUCs of the three risk factors are presented in Fig. 2; Table 6. In the 5- and 10-year follow-up groups, LAP had the largest AUC (0.644 and 0.651, respectively) followed by TyG (0.608 and 0.617, respectively) and VAI (0.591 and 0.595, respectively). In the 5-year follow-up, the optimal cut-off points for LAP, TyG, and VAI were found to be 34.38, 8.47, and 2.36, respectively. For the 10-year follow-up, the optimal cut-off points were 28.2 for LAP, 8.99 for TyG, and 2.63 for VAI. The results of the stratified analysis, as presented in Supplementary Tables 3, align with the total population analysis.

**Table 6:** Diagnostic performance of LAP, TyG, and VAI in detecting CVD

Total	AUC (95% CI)	Cut-off	Sensitivity	Specificity	Youden index
5-year incidence					
WC	0.655 (0.624, 0.685)	90	47	76	0.232
LAP	0.644 (0.615, 0.673)	34.38	65	60	0.249
BMI	0.617 (0.586, 0.647)	24.83	69	50	0.188
TyG	0.608 (0.578, 0.639)	8.47	74	44	0.174
VAI	0.591 (0.561, 0.622)	2.36	54	60	0.143
TG	0.587 (0.557, 0.618)	95	78	37	0.145
FBS	0.577 (0.544, 0.610)	102	33	79	0.123
TC	0.571 (0.539, 0.603)	174	74	40	0.133
LDL	0.561 (0.529, 0.505)	122	60	51	0.112
HDL	0.470 (0.437, 0.503)	20	99	02	0.004
10-year incidence					
LAP	0.651 (0.622, 0.681)	28.21	71	53	0.249
WC	0.649 (0.619, 0.679)	81	72	51	0.232
BMI	0.633 (0.602, 0.663)	25.35	64	56	0.209
TyG	0.617 (0.586, 0.626)	8.99	40	79	0.196
TC	0.612 (0.581, 0.642)	174	77	42	0.195
TG	0.605 (0.573, 0.636)	99	75	41	0.159
LDL	0.595 (0.564, 0.626)	114.2	74	44	0.179
VAI	0.595 (0.563, 0.626)	2.63	48	69	0.170
FBS	0.593 (0.561, 0.625)	97	43	72	0.149
HDL	0.487 (0.455, 0.519)	37	52	49	0.007

AUC, area under curve; BMI, body mass index; CVD, cardiovascular disease; FBS, fasting blood sugar; HDL, high-density lipoprotein; LAP, lipid accumulation product; LDL, low-density lipoprotein; TC, total cholesterol; TG, triglyceride; TyG, triglyceride-glucose index; VAI, visceral adiposity index, WC, waist circumference

The ROC curve of novel and traditional risk factors is presented in Fig. 2 and Supplementary Figs. 1 and 2. In both the 5-year and 10-year follow-ups, LAP consistently ranked top among the risk factors for predicting cardiovascular outcomes in both males and females. TyG was in the next rank. VAI showed varying rankings across different follow-up periods and sexes. Remarkably, single lipid profiles and FBS showed almost the poorest performance among the evaluated risk factors in the 5-year and 10-year follow-ups.

## Discussion

The findings of this study showed a significant association between LAP, TyG, VAI, and CVD odds. Furthermore, as there are currently no established optimal cut-off points for LAP and VAI, our research findings provided a basis for future studies in this area. Previous works exploring the association between these three indices and the odds of CVD have yielded inconsistent results, and the exact nature of the relationship remains unclear. The studies conducted in Argentina [14] and China [15] did not find significant associations between TyG and LAP and CVD risk. Furthermore, Bozorgmanesh et al. reported no association between LAP [3] and VAI [6], and CVD risk in men. No previous study has evaluated the effectiveness of LAP, TyG, and VAI comparatively in predicting CVD risk.

LAP is a reflection of the underlying continuous process that occurs with the excessive deposition of visceral fat, which progressively leads to metabolic dysregulation, low-grade inflammation, and atherosclerosis. For example, in the TLGS study comprising 6751 participants, the LAP index was associated with 1.41 times higher risk of CVD in women [3]. Another study from TLGS with 2378 participants showed that people in the 3rd tertile had 2.17 times higher risk of CVD development compared with the 1st tertile [16]. Data from 3042 Greek adults also showed that a 10-unit increase in LAP was associated with an 11% increase in CVD risk [17]. A six-year follow-up of 7837 participants in southwest China reported 1.19 times higher risk of CVD incidents in females but not in males [15]. These sex differences might be related to sex hormones, adiposity distribution, and higher rates of diabetes and low physical activity in females, which are related to development of insulin resistance (IR). In the present study, the analysis of ROC curves revealed a threshold value of 34.4 for LAP in the detection of CVD, with a sensitivity of 65%, specificity of 60%, and AUC of 0.644. This AUC was larger than that reported in a Chinese study (men: 0.498 [0.455–0.541], women: 0.563 [0.527–0.60]) [15]. In another Iranian study (men: 0.785 [0.756–0.813], women: 0.846 [0.818–0.875]) [3], and a in a Caucasian study (total 0.80 [0.77–0.83]) [17] larger AUCs were also reported. It seems that the ethnicity is a factor affecting AUC for LAP, as the east Asian populations (China, Korea) have lower AUCs than Caucasian populations (Iran, Greece). Regarding the comparison between traditional single risk factors and the LAP index, in both of our follow-ups, the AUC of LAP was greater than the AUC for traditional risk factors, with the exception of the WC in the 10-year follow-up.

Based on our analysis, we have identified a TyG value of 8.47 as the cut-off point for CVD in our study population, with an AUC of 0.608, sensitivity of 84%, and specificity of 44%. A recent cohort study conducted on 141,243 participants from five continents showed that high TyG was associated with a greater incidence of CVD [18]. Liu et al. also conducted a cohort data analysis and found that participants with higher TyG were at higher risk for CVD and CHD [19]. Similar results were observed in the general population of China [20], Korea [21] and united kingdom [22]. In Iran, two studies have shown that TyG is significantly associated with an increased risk of CVD incidence [4, 10], and another study has shown an association between TyG-BMI and hypertension [23]. Salazar et al. studied 723 individuals and showed that high TyG did not increase the odds of CVD [14]. The discrepancies can be attributed to their exclusion of individuals with diabetes, who typically have higher TyG values. It is noteworthy that our cut-off point is lower than the cut-off point identified in the TLGS [4], which was 9.02 (with a sensitivity of 59.23% and specificity of 63.15%). However, it is close to the cut-off point reported in the Korean Cohort [21], which was 8.6. In the study by Liu et al., the AUC of TyG was reported to be 0.730 (0.706–0.754) [19], and Korean study reported even a lower AUC of 0.578 [21]. The lower AUC in the last two studies may be due to the assessing association between the TyG index and risk of CVD in non-diabetic population [19] or adjustment for diabetes in the analysis [21]. Regarding the comparison between traditional single risk factors and the TyG index, in both of our follow-ups, the AUC of TyG surpassed the AUC for the traditional single risk factors, except for WC and BMI.

VAI considers both the metabolic/lipidemic (TG and HDL) and anthropometric (BMI and WC) variables related to obesity, which may suggest that it more accurately reflects the potential pro-atherogenic processes. Our results showed that the AORs for CVD risk in the 5- and 10- year follow ups in the 4th quantile were 58% and 41% higher than the 1st quartile, respectively. In a nine-year follow-up of 6407 participants, Bozorgmanesh et al. found that VAI was a risk factor for future CVD in the females, but not in males [6]. Moreover, the studies conducted on 3042 people in the Athens metropolitan region in Greece (ATTICA) found that each ten-unit VAI increase was associated with 5% higher 10-year CVD incidence [24]. In the RaND study, conducted on 7362 Iranian adults, it was found that being in tertile 3 of VAI is associated with a 1.25-fold risk of CVD compared with tertile 1 [7]. In the present study, VAI exhibited the poorest performance in predicting CVD risk among the three combinational risk factors. The AUC for VAI was 0.591, with a cut-off value of 2.36, resulting in a sensitivity of 54% and a specificity of 60%. Nevertheless, during the 5-year follow-up, VAI still exhibited superior performance in AUC compared to the traditional single risk factors.

### Strengths and limitations

The present study has some strengths and limitations. The strengths include long-term follow-up, which provides better insights over an extended period, a relatively large sample size, which enhances the statistical robustness of the findings, and adjustment for several confounders, which improves the accuracy of analyses. Furthermore, comparing the predicting power of the three risk factors in one study along with comparison with the predicting power of traditional single risk factors for the incidence of CVD are among the strengths. Meanwhile, there are some limitations as well: The study population was homogenous and included people from urban areas, which means caution should be exercised when generalizing the findings to the other geographical populations and rural areas. Secondly, we were unable to account for changes in components of combinational risk factors that may have happened over time, as our analysis was based on their baseline values.

## Conclusion

Overall the study suggests that LAP, TyG, and VAI predict the risk of CVD in the southeastern population of Iran. Using these three combinational risk factors instead of simple measures of lipids may provide more reliable results in predicting CVD risk over 5- and 10-year follow ups. LAP seems to be a stronger indicator of CVD risk than TyG and VAI. Longer follow ups comprising both urban and rural populations will show whether monitoring LAP, TyG, and VAI can still be a reliable method for CVD risk prediction.

### Electronic supplementary material

Below is the link to the electronic supplementary material.

Supplementary Material 1

## Data Availability

The datasets used are available from the corresponding author on reasonable request.
