# Supplementary material for: Evaluation of the novel three lipid indices for predicting five- and ten-year incidence of cardiovascular disease: findings from Kerman coronary artery disease risk factors study (KERCADRS)
Source: Lipids Health Dis. 2023 Oct 5;22:169. doi: 10.1186/s12944-023-01932-x (PMC10552300; doi:10.1186/s12944-023-01932-x)
Supplement: Supplementary file 1 — Supplementary Material 1 [file 12944_2023_1932_MOESM1_ESM.docx]

**Supplementary file**

**Title: Evaluation of the novel three lipid indices for predicting five- and ten-year incidence of cardiovascular disease: Findings from Kerman Coronary Artery Disease Risk Factors Study (KERCADRS)**

**Supplementary Table 1.** Comparison of baseline characteristics of participants according to sex in the **5-year follow-up group**.

| Subgroups | Total | Male | | Female | |
| --- | --- | --- | --- | --- | --- |
|  | **(n=1888)** | **No-CVD group (n=632)** | **CVD group (n=183)** | **No-CVD group (n=857)** | **CVD group (n=216)** |
| Age, mean (SD) | 54.06 (14.02) | 53.33 (14.86) | 62.15 (14.01) ^†^ | 51.15 (12.77) | 60.94 (11.09) ^†^ |
| Cigarette smoking, n (%) | 200 (10.6) | 149 (23.6) | 41 (22.4) | 7 (0.82) | 3 (1.39) |
| Education (University) n (%) | 368 (19.51) | 145 (22.94) | 46 (25.14) | 152 (17.78) | 25 (11.57) |
| Low physical activity n (%) | 801 (42.43) | 243 (38.45) | 69 (37.70) | 384 (44.81) | 105 (48.61) |
| BMI (kg/m^2^) | 25.47 (4.79) | 23.87 (4.11) | 25.92 (4.39) ^†^ | 25.96 (5.00) | 27.84 (4.67) ^†^ |
| Waist circumference (cm) | 83.57 (11.96) | 84.66 (11.28) | 91.07 (11.44) ^†^ | 80.34 (11.57) | 86.80 (11.57) ^†^ |
| FBS (mg/dl) | 97.78 (30.79) | 97.65 (27.18) | 101.95 (28.95) | 94.71 (28.21) | 106.74 (46.20) ^†^ |
| TC (mg/dl) | 191.82 (41.94) | 188.72 (41.98) | 192.79 (38.01) | 190.70 (41.69) | 204.51 (43.89) ^†^ |
| TG (mg/dl) | 140.96 (88.40) | 146.58 (99.29) | 161.99 (92.80) | 129.59 (79.89) | 151.71 (76.36) ^†^ |
| HDL-C (mg/dl) | 38.75 (9.57) | 36.56 (9.28) | 34.48 (8.13) ^†^ | 40.74 (9.41) | 40.87 (9.60) |
| LDL-C (mg/dl) | 125.42 (35.03) | 123.57 (35.95) | 126.50 (33.32) | 124.32 (34.42) | 134.34 (34.96) ^†^ |
| SBP (mm Hg) | 109.48 (12.57) | 111.19 (10.98) | 116.72 (10.21) ^†^ | 105.32 (12.70) | 114.91 (12.23) ^†^ |
| DBP (mm Hg) | 73.78 (7.08) | 74.12 (7.05) | 77.39 (5.30) ^†^ | 72.43 (7.18) | 75.09 (6.66) ^†^ |
| LAP | 39.57 (36.23) | 37.09 (37.93) | 50.18 (38.33) ^†^ | 36.04 (32.84) | 51.85 (37.97) ^†^ |
| TyG | 8.66 (0.62) | 8.70 (0.61) | 8.85 (0.59) ^†^ | 8.55 (0.62) | 8.83 (0.60) ^†^ |
| VAI | 2.85 (2.81) | 2.68 (3.12) | 3.07 (2.41) | 2.81 (2.67) | 3.33 (2.36) ^†^ |
| CVD, cardiovascular disease; FBS, fast blood glucose; TC, total cholesterol; TG, triglyceride; HDL, high-density lipoprotein; LDL, low-density lipoprotein; SBP, systolic blood pressure; DBP, diastolic blood pressure; LAP, lipid accumulation product; TyG, triglyceride glucose index; VAI, visceral adiposity index.  Data are presented as Mean (SD) or Median [P25-75] for continuous measures, and n (%) for categorical measures.  ^†^*P* < 0.05 using ANOVA test. | | | | | |

**Supplementary Table 2.** Comparison of baseline characteristics of participants according to sex in the **10-year follow-up group**.

| Subgroups | Total | Male | | Female | |
| --- | --- | --- | --- | --- | --- |
|  | **(n=1450)** | **No-CVD group (n=420)** | **CVD group (n=209)** | **No-CVD group (n=554)** | **CVD group (n=267)** |
| Age (years) | 53.52 (13.09) | 51.87 (13.53) | 69.37 (12.74) ^†^ | 49.77 (12.03) | 59.30 (10.99) ^†^ |
| Cigarette smoking n (%) | 200 (10.59) | 88 (20.95) | 51 (24.40) | 5 (0.90) | 3 (1.12) |
| Education (University) n (%) | 368 (19.51) | 160 (27.38) | 49 (23.35) ^†^ | 101 (18.27) | 31 (11.66) ^†^ |
| Low physical activity n (%) | 801 (42.43) | 138 (32.86) | 91 (43.54) ^†^ | 258 (46.57) | 122 (45.69) |
| BMI (kg/m^2^) | 25.47 (4.79) | 24.07 (4.35) | 25.74 (3.87) ^†^ | 25.62 (5.09) | 27.80 (4.47) ^†^ |
| Waist circumference (cm) | 83.57 (11.96) | 85.02 (11.82) | 89.84 (10.60) ^†^ | 79.27 (11.48) | 86.01 (10.91) ^†^ |
| FBS (mg/dl) | 97.78 (30.79)  92 [83, 101] | 95.38 (21.14)  92 [85, 101] | 101.03 (27.04) ^†^  95 [88, 106] | 93.34 (28.35)  89 [80.5, 97] | 101.85 (34.23) ^†^  94 [85, 108] |
| TC (mg/dl) | 191.82 (41.94) | 186.25 (42.37) | 199.33 (38.51) ^†^ | 188.03 (40.87) | 204.01 (41.21) ^†^ |
| TG (mg/dl) | 140.96 (88.40)  119 [85, 169] | 145.02 (96.64)  124 [85, 168] | 170.04 (101.42) ^†^  148 [101, 208] | 121.78 (67.92)  106 [76, 147] | 157.57 (96.14) ^†^  131 [96, 188] |
| HDL-C (mg/dl) | 38.75 (9.57) | 36.13 (8.89) | 34.75 (8.42) | 40.89 (9.34) | 40.89 (9.73) |
| LDL-C (mg/dl) | 125.42 (35.03) | 121.83 (36.20) | 131.17 (34.91) ^†^ | 122.86 (34.30) | 133.35 (32.89) ^†^ |
| SBP (mm Hg) | 109.48 (12.57) | 110.61 (10.91) | 117.26 (11.94) ^†^ | 104.49 (13.17) | 113.65 (12.08) ^†^ |
| DBP (mm Hg) | 73.78 (7.08) | 74.00 (7.11) | 77.15 (6.63) ^†^ | 71.90 (7.54) | 74.72 (6.74) ^†^ |
| LAP | 39.57 (36.23) | 36.81 (36.71) | 51.72 (42.29) ^†^ | 32.55 (29.63) | 52.29 (40.87) ^†^ |
| TyG | 8.66 (0.62) | 8.67 (0.59) | 8.88 (0.61) ^†^ | 8.49 (0.57) | 8.81 (0.66) ^†^ |
| VAI | 2.85 (2.81) | 2.65 (2.92) | 3.23 (3.00) ^†^ | 2.58 (2.10) | 3.52 (3.28) ^†^ |
| CVD, cardiovascular disease; FBS, fast blood glucose; TC, total cholesterol; TG, triglyceride; HDL, high-density lipoprotein; LDL, low-density lipoprotein; SBP, systolic blood pressure; DBP, diastolic blood pressure; LAP, lipid accumulation product; TyG, triglyceride glucose index; VAI, visceral adiposity index.  Data are presented as Mean (SD) or Median [P25-75] for continuous measures, and n (%) for categorical measures.  ^†^*P* < 0.05 using ANOVA test. | | | | | |

**Supplementary Table** **3.** Diagnostic performance of LAP, TyG, and VAI in detecting gender-specific 5- and 10-year incidence of cardiovascular disease (CVD)

| Total | AUC (95% CI) | Cut-off | Sensitivity | Specificity | Youden index |
| --- | --- | --- | --- | --- | --- |
| 5-year incidence, Male | | | | | |
| WC | 0.660 (0.615, 0.706) | 92 | 50 | 75 | 0.254 |
| LAP | 0.632 (0.587, 0.677) | 31.40 | 69 | 65 | 0.252 |
| BMI | 0.631 (0.585, 0.677) | 24.14 | 68 | 51 | 0.191 |
| TyG | 0.567 (0.519, 0.614) | 8.47 | 76 | 38 | 0.148 |
| VAI | 0.577 (0.530, 0.624) | 2.42 | 50 | 64 | 0.144 |
| TG | 0.556 (0.509, 0.604) | 95 | 80 | 33 | 0.128 |
| FBS | 0.554 (0.503, 0.604) | 96 | 48 | 63 | 0.113 |
| TC | 0.537 (0.489, 0.583) | 175 | 69 | 43 | 0.124 |
| LDL | 0.531 (0.483, 0.579) | 121 | 60 | 50 | 0.099 |
| HDL | 0.438 (0.391, 0.485) | 20 | 98 | 03 | 0.008 |
| 5-year incidence, Female | | | | | |
| WC | 0.651 (0.610, 0.692) | 90 | 40 | 82 | 0.225 |
| LAP | 0.655 (0.615, 0.694) | 33.23 | 67 | 59 | 0.260 |
| BMI | 0.613 (0.572, 0.653) | 24.92 | 76 | 44 | 0.202 |
| TyG | 0.635 (0.595, 0.675) | 8.69 | 58 | 62 | 0.198 |
| VAI | 0.600 (0.559, 0.641) | 2.74 | 49 | 66 | 0.152 |
| TG | 0.612 (0.571, 0.652) | 111 | 66 | 52 | 0.173 |
| FBS | 0.592 (0.548, 0.637) | 104 | 32 | 83 | 0.147 |
| TC | 0.598 (0.556, 0.641) | 171 | 80 | 35 | 0.150 |
| LDL | 0.584 (0.541, 0.628) | 124.4 | 61 | 53 | 0.139 |
| HDL | 0.503 (0.456, 0.546) | 39 | 55 | 48 | 0.036 |
| 10-year incidence, Male | | | | | |
| LAP | 0.633 (0.587, 0.680) | 32.29 | 66 | 58 | 0.231 |
| WC | 0.631 (0.585, 0.678) | 84 | 72 | 51 | 0.230 |
| BMI | 0.626 (0.580, 0.672) | 24.43 | 68 | 53 | 0.213 |
| TyG | 0.602 (0.554, 0.650) | 9.08 | 42 | 78 | 0.199 |
| TC | 0.603 (0.556, 0.649) | 168 | 78 | 40 | 0.183 |
| TG | 0.592 (0.543, 0.640) | 152 | 47 | 67 | 0.144 |
| LDL | 0.588 (0.541, 0.636) | 112.4 | 73 | 43 | 0.163 |
| VAI | 0.591 (0.543, 0.639) | 2.63 | 47 | 70 | 0.173 |
| FBS | 0.578 (0.529, 0.627) | 102 | 33 | 79 | 0.127 |
| HDL | 0.456 (0.407, 0.505) | 19 | 98 | 02 | 0.002 |
| 10-year incidence, Female | | | | | |
| LAP | 0.665 (0.627, 0.704) | 28.21 | 73 | 54 | 0.271 |
| WC | 0.666 (0.627, 0.705) | 82 | 63 | 63 | 0.252 |
| BMI | 0.644 (0.604, 0.683) | 26.10 | 67 | 58 | 0.242 |
| TyG | 0.629 (0.587, 0.670) | 8.47 | 72 | 50 | 0.215 |
| TC | 0.619 (0.579, 0.659) | 175 | 79 | 43 | 0.221 |
| TG | 0.616 (0.574, 0.657) | 109 | 66 | 53 | 0.196 |
| LDL | 0.600 (0.559, 0.641) | 114.2 | 76 | 43 | 0.195 |
| VAI | 0.599 (0.556, 0.640) | 2.33 | 57 | 60 | 0.177 |
| FBS | 0.603 (0.561, 0.646) | 97 | 42 | 76 | 0.179 |
| HDL | 0.513 (0.470, 0.556) | 37 | 66 | 39 | 0.047 |
| AUC, area under curve; BMI, body mass index; CVD, cardiovascular disease; FBS, fast blood sugar; HDL, high-density lipoprotein; LAP, lipid accumulation product; LDL, low-density lipoprotein; TC, total cholesterol; TG, triglyceride; TyG, triglyceride-glucose index; VAI, visceral adiposity index, WC, waist circumference. | | | | | |


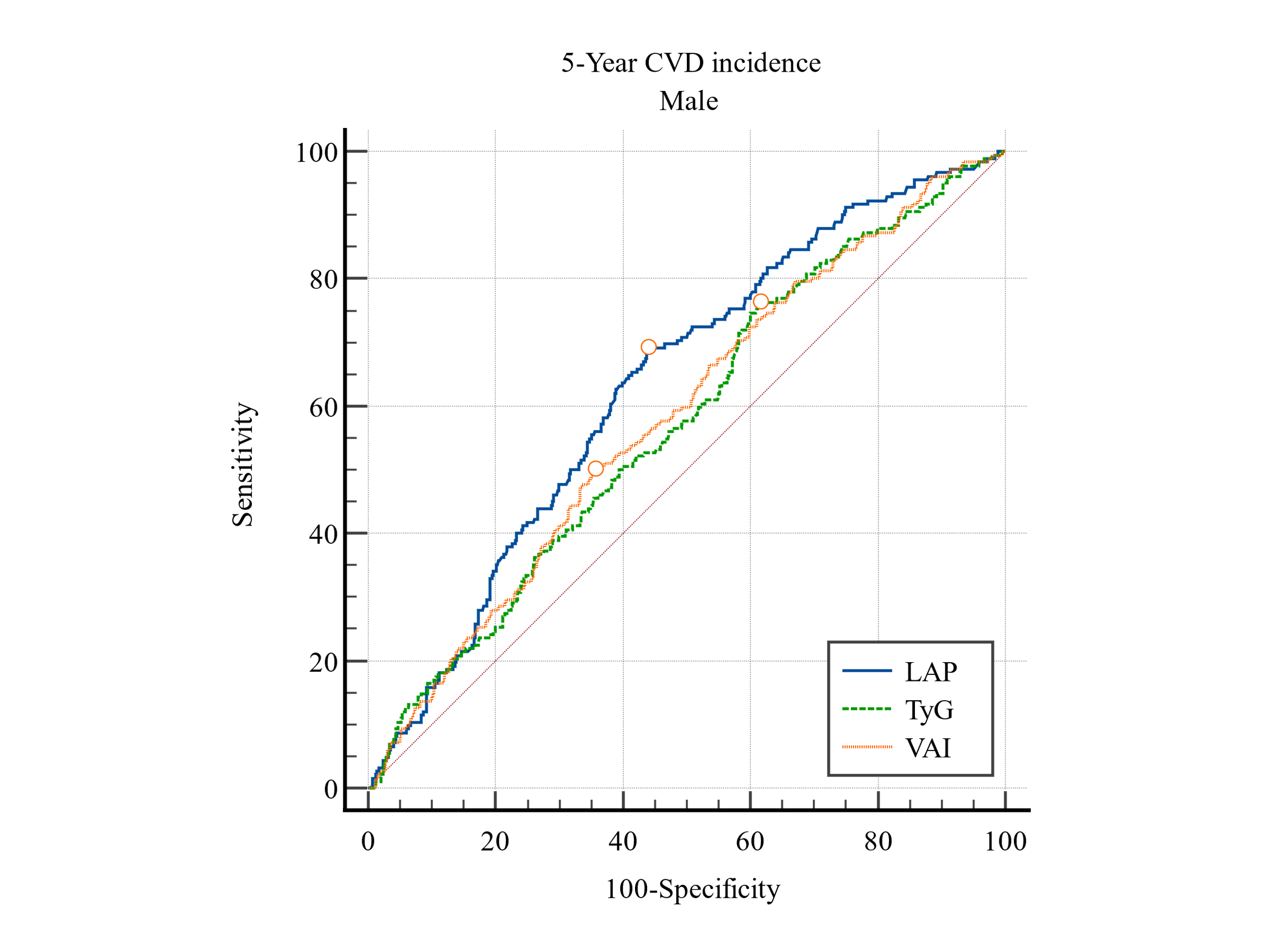

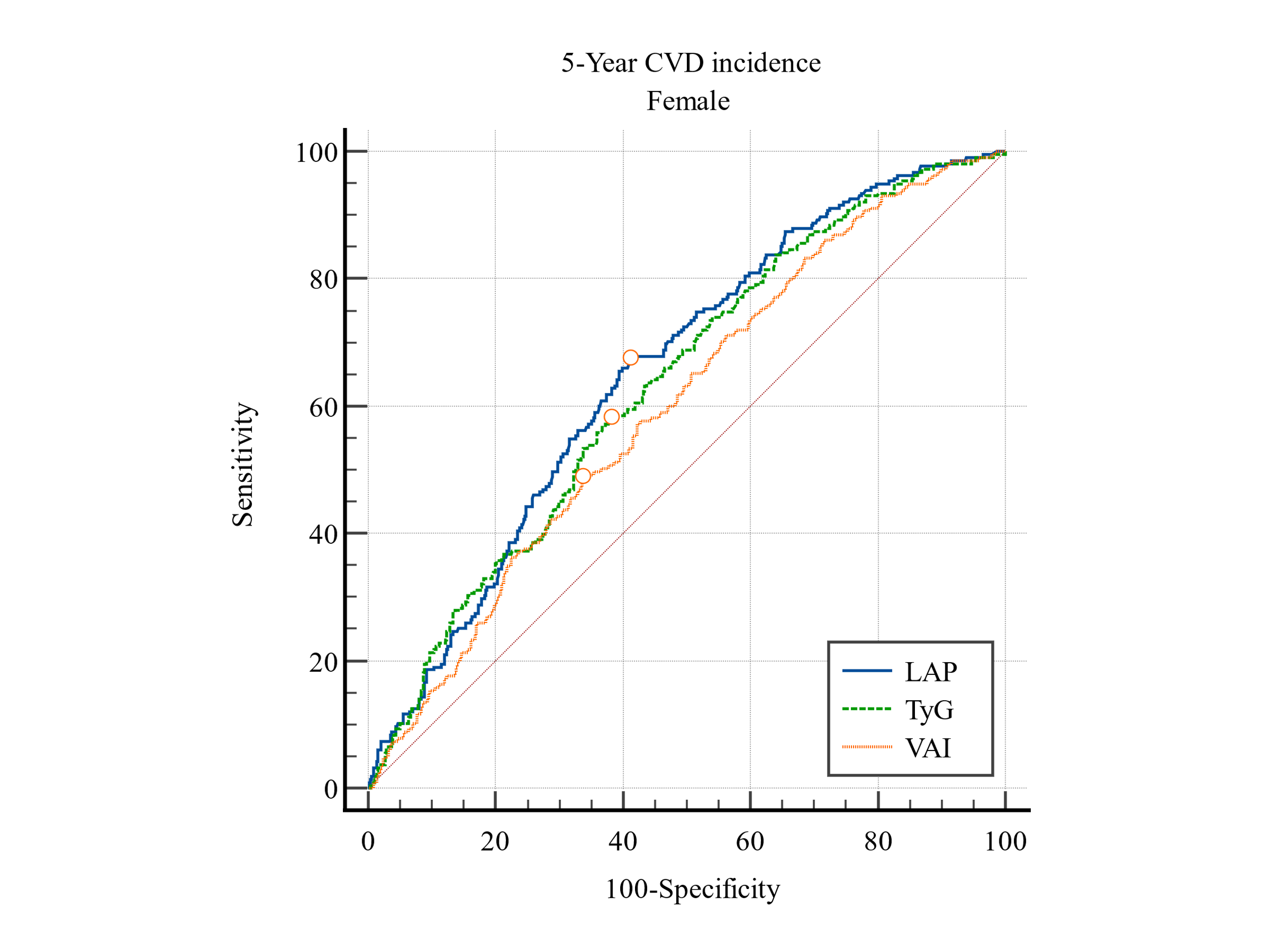


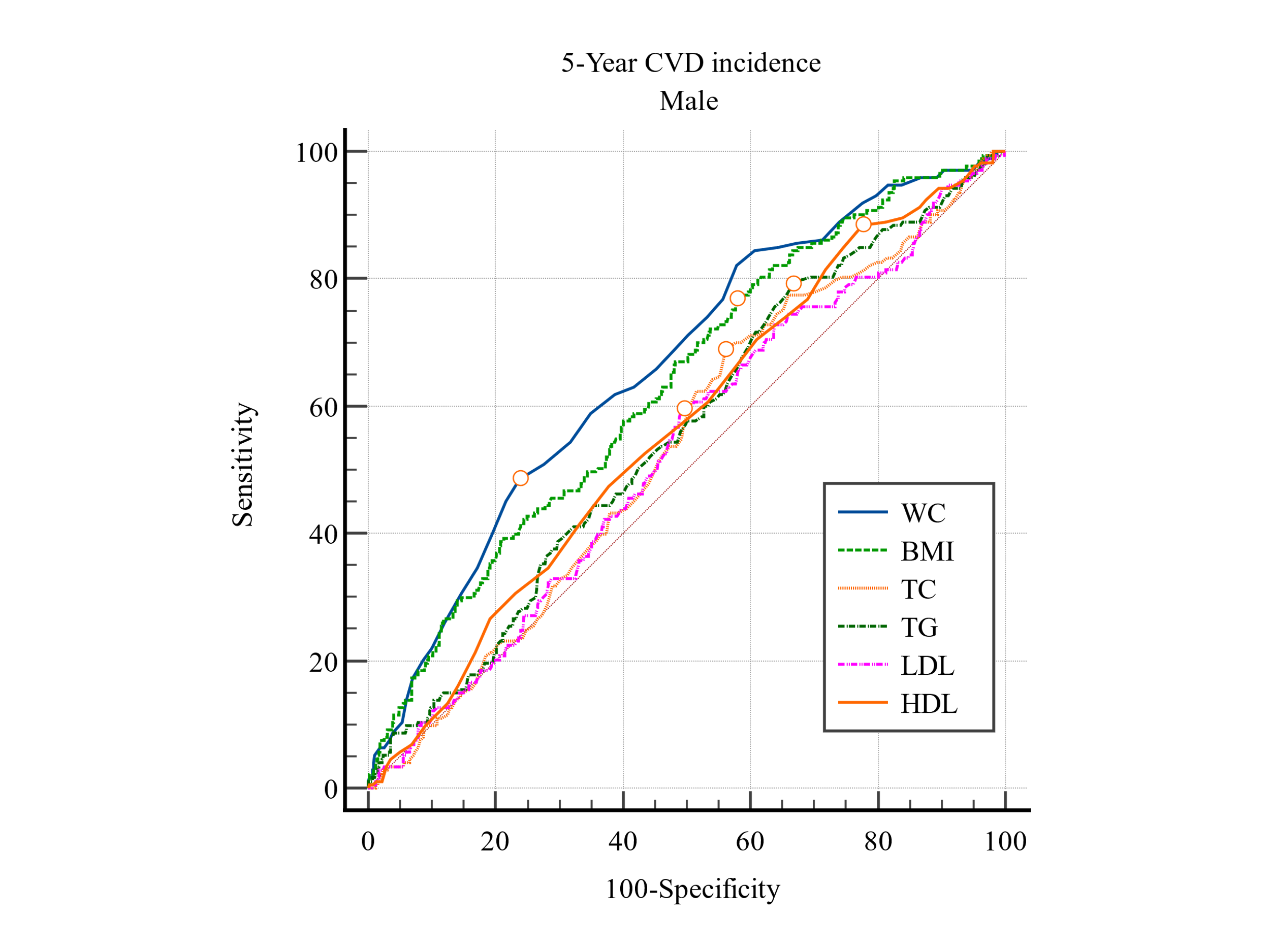

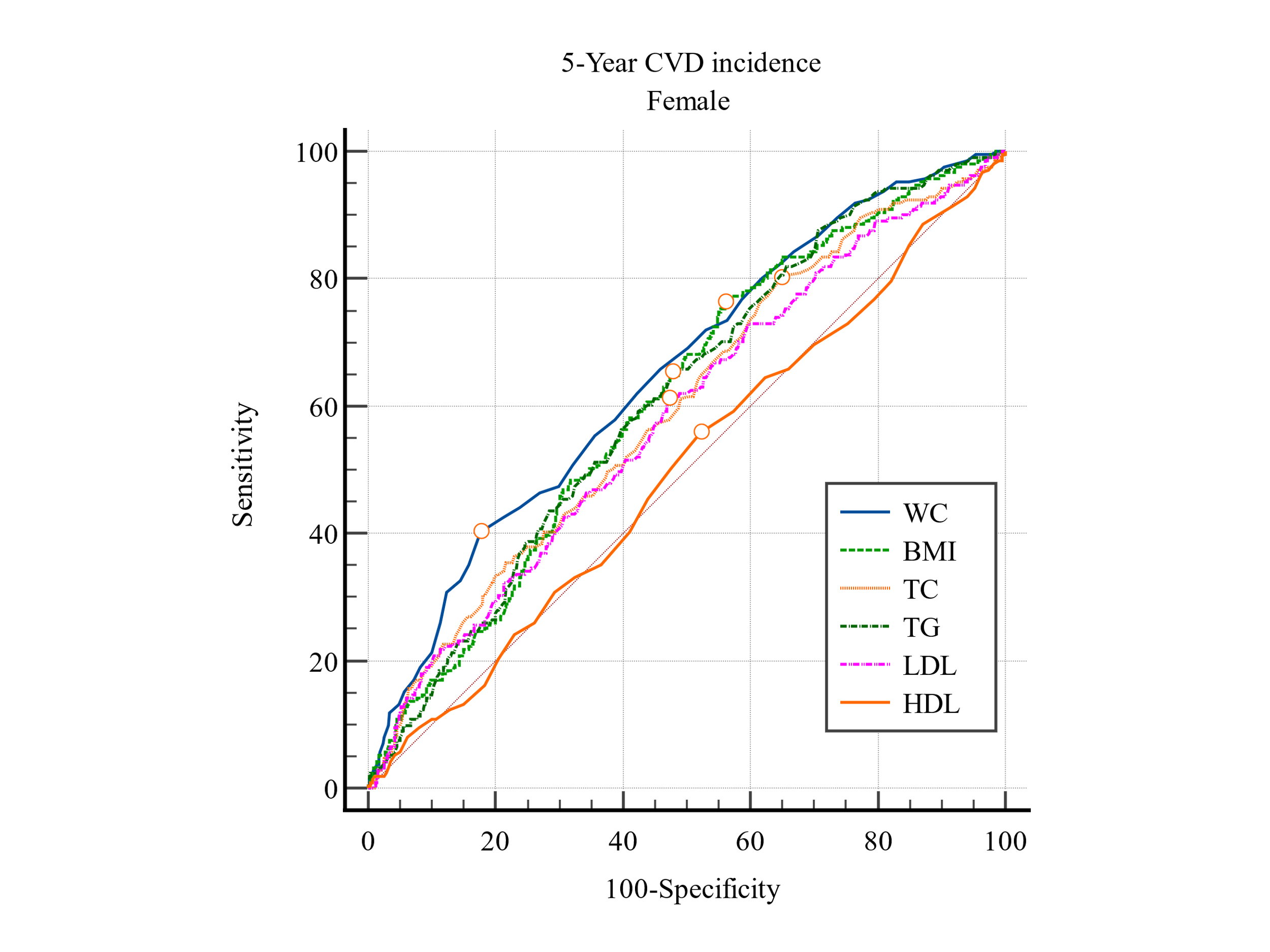


**Supplementary figure 1.** Comparison of receiver operating characteristic (ROC) curves of new (upper panels) and traditional (lower panels) CVD risk factors of 5-year cardiovascular disease (CVD) incidence in male (left) and female (right)


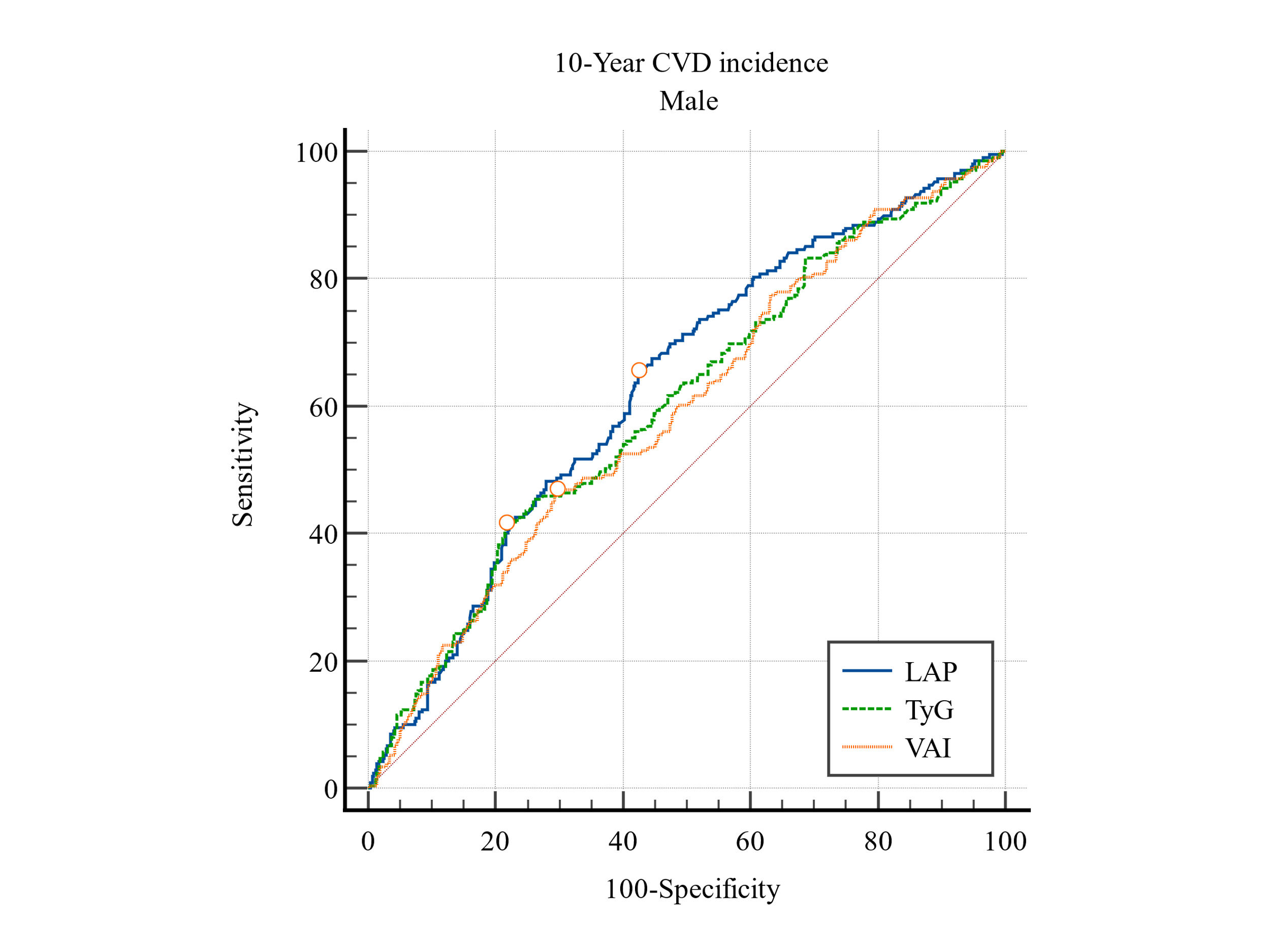

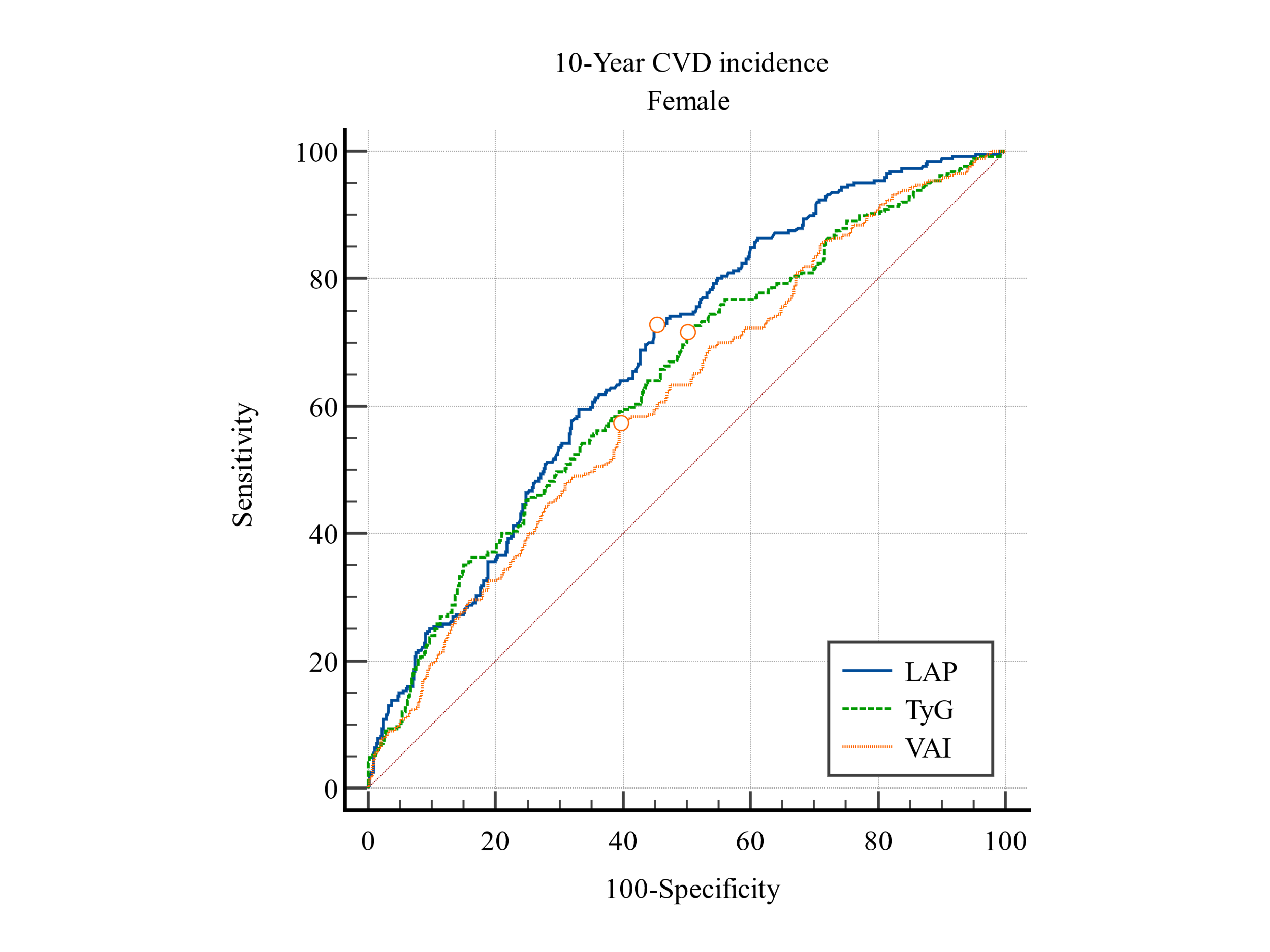

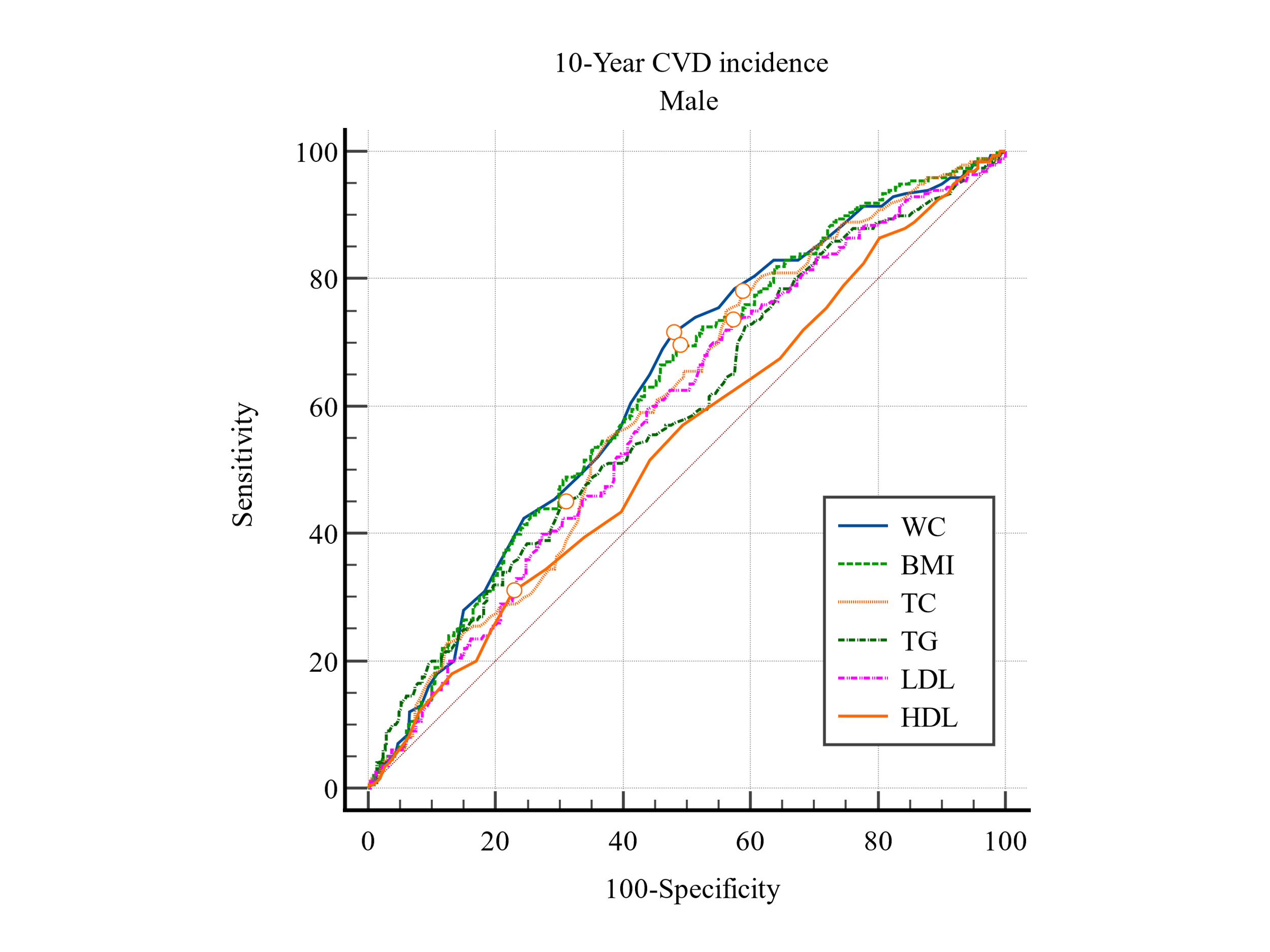

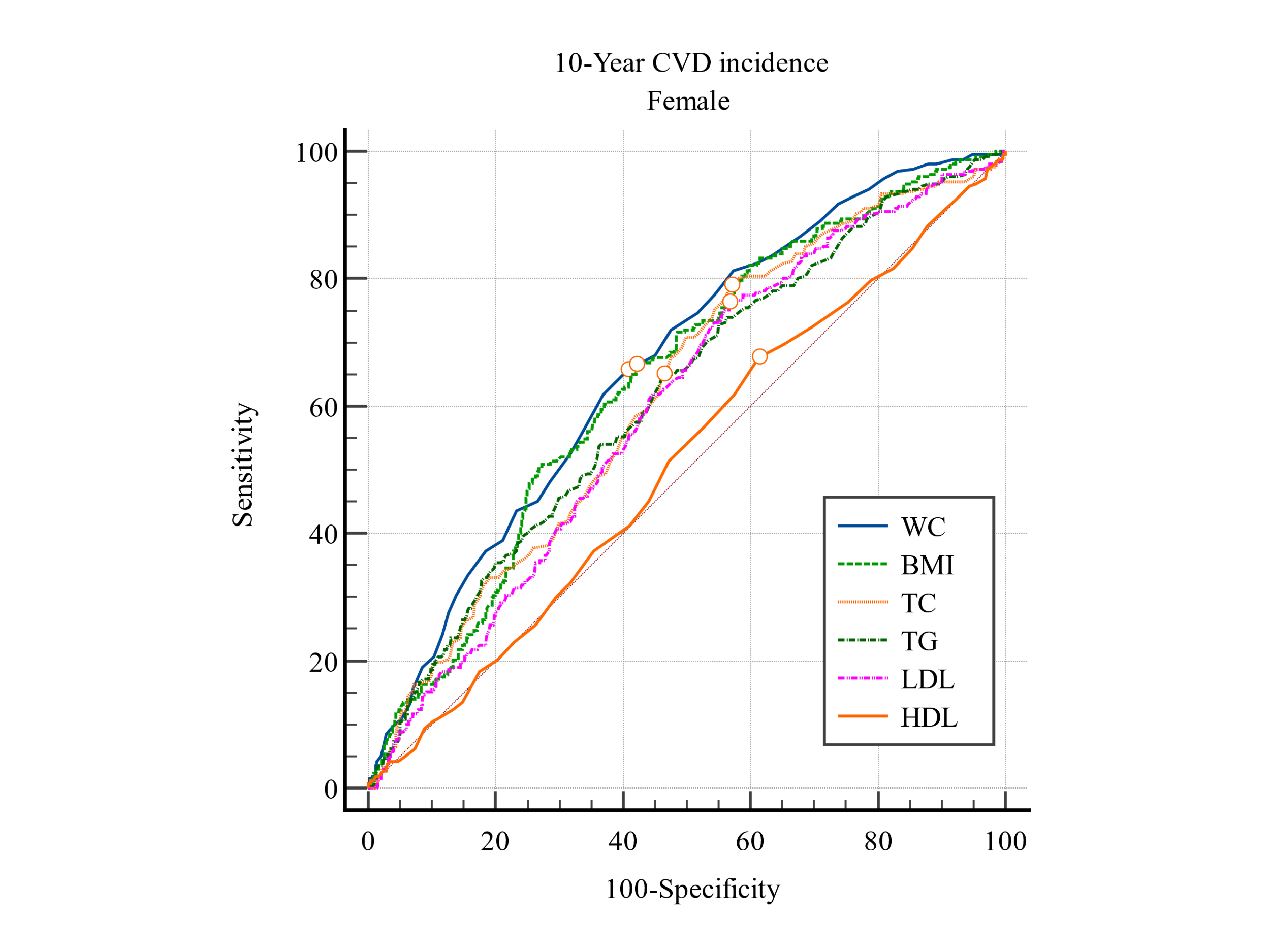


**Supplementary Figure 2.** Comparison of receiver operating characteristic (ROC) curves of new (upper panels) and traditional (lower panels) CVD risk factors of 10-year cardiovascular disease (CVD) incidence in male (left) and female (right)
